# Application of a fast and cost-effective ‘three-in-one’ MMR ELISA as a tool for surveying anti-MMR humoral immunity: the Hungarian experience

**DOI:** 10.1017/S0950268819002280

**Published:** 2020-02-04

**Authors:** K. Böröcz, Z. Csizmadia, Á. Markovics, N. Farkas, J. Najbauer, T. Berki, P. Németh

**Affiliations:** 1 Department of Immunology and Biotechnology, Clinical Centre, University of Pécs Medical School, Pécs, Hungary; 2 Department of General and Physical Chemistry, Faculty of Natural Sciences, University of Pécs, Pécs, Hungary; 3 Department of Bioanalysis, University of Pécs Medical School, Pécs, Hungary

**Keywords:** IgG, immunity, indirect ELISA, measles, MMR, mumps, rubella, vaccine

## Abstract

In Hungary, between February 2017 and July 2019, 70 confirmed measles cases were reported, raising questions about the adequacy of population-level immunity. Although the assumed vaccination coverage is ≥99%, in a recent study, we detected potential gaps in the anti-measles humoral immunity. In Hungary, according to a decree by the Ministry of Public Welfare, beginning from 2021, the healthcare provider should conduct a serosurvey of anti-measles protection levels of healthcare professionals. To facilitate the compliance with this requirement, we developed a quick ‘three-in-one’ or ‘triple’ MMR (measles, mumps and rubella) indirect ELISA (IgG); an assay format that is currently not available commercially. High throughput applicability of the ‘three-in-one’ ELISA was verified using 1736 sera from routine laboratory residual samples, using an automated platform (Siemens BEP 2000 Advance). Assay verification was performed by comparing the full antigen repertoire-based ‘target’ assay with in-house ‘control’ assays using recombinant viral antigen coatings, and by validated commercially available kits. Indirect immunofluorescence was used as an independent reference method. Data were analysed using OriginLab, IBM SPSS, RStudio and MedCalc. In case of measles, we combined our current results with previously published data (*N*_total measles_ = 3523). Evaluation of anti-mumps and anti-rubella humoral antibody levels was based on the measurement of 1736 samples. The lowest anti-measles seropositivity (79.3%) was detected in sera of individuals vaccinated between 1978 and 1987. Considering the antigen-specific seropositivity ratios of all samples measured, anti-measles, -mumps and -rubella IgG antibody titres were adequate in 89.84%, 91.82% and 92.28%, respectively. Based on the virus-specific herd immunity threshold (HIT) values (HIT_Measles_ = 92–95%, HIT_Mumps_ = 75–86%, HIT_Rubella_ = 83–86), it can be stated that regarding anti-measles immunity, certain age clusters of the population may have inadequate levels of humoral immunity. Despite the potential gaps in herd immunity, the use of MMR vaccine remains an effective and low-cost approach for the prevention of measles, mumps and rubella infections.

## Introduction

Despite the existence of effective measles (M) and measles-containing vaccines (MCV), resurgence of measles cases in the USA and across Europe has occurred, including individuals vaccinated with two doses of the vaccine [1]. In Europe, a safe and effective two-dose vaccination schedule has been made available since the 1960s. The introduction of the trivalent measles, mumps, rubella (MMR) vaccines started in the 1970s [2] (in Hungary in 1991, Fig. 1), and it is still in practice, in the form of modern and safe tri- and tetravalent (measles, mumps, rubella and varicella; MMRV) vaccines. However, the risk of continued widespread circulation of measles in EU/EEA still exists, since significant immunity gaps persist, due to suboptimal historical and current vaccination coverage [3]. Despite regional outbreaks of measles infections, in 2016, globally fewer than 1 00 000 individuals died from measles, as a result of recent improvements of national immunisation programmes. In the WHO European Region (WHO EUR), between 2009 and 2017, the estimated regional coverage was 93–95% for the first dose of measles-containing vaccines (MCV1), and increased from 73% to 90% for the second dose (MCV2) [4]. In spite of the improving vaccine coverage tendencies, measles incidence increased again to 89.5 per 1 million population in 2018 in the EU region [4]. From 1 July 2018 to 30 June 2019, 30 EU/EEA Member States reported 13 102 cases of measles, also including fatalities [5]. According to WHO reports, the largest outbreaks occur in countries with low measles vaccination coverage. However, outbreaks occurred even in countries with high national vaccination rates [6]. Lately, an alarming surge of measles cases was experienced in countries neighbouring Hungary. From 2017 to 2018, Ukraine had the largest increase in measles cases worldwide [7, 8]. In 2018, Ukraine reported >54 000 measles cases; more than the entire EU. The total estimated number of measles cases for the first 5 months of 2019 was 52 034, including 17 deaths [9, 10]. Romania also bears a high burden of the disease; between the first outbreak (late 2016) and May 2019, Romania has reported 16 627 cases and 63 deaths. Ninety-four per cent of the reported cases were unvaccinated individuals, and 4% received only one of the two-shot vaccination series. Regarding parotitis epidemica (mumps), the last accessible ECDC surveillance report is from 2016; 28 EU/EEA countries reported 14 795 cases of mumps, of which the Czech Republic, Poland, Spain and the UK were responsible for 77% of these cases. The mumps childhood vaccination coverage in Hungary is ≥99% (MCV1 and MCV2 are equivalent to MMR1 and MMR2 in Supplementary Fig. S1. Supplementary materials are available on the Cambridge Core website), consequently, the risk of infection is predominantly by virus importation [11]. In Hungary, the rubella vaccine was introduced in 1990 in the form of measles–rubella (MR) bivalent vaccine. A year later, in 1991, it was replaced by the MMR trivalent vaccine that is still in use today. From 1 July 2018 to 30 June 2019, EU/EEA Member States reported 483 cases of rubella. The highest number of cases was reported by Poland (372), Germany (57), Italy (24), Spain (12) and Romania (4) [5]. For Hungary, between 2007 and 2018, WHO reports only 10 cases [12]. Measles, mumps and rubella statistics (cases per year) based on WHO measles and rubella ‘*elimination country profile for Hungary*’ data (i.e. the number of reported infections of the last decades) are shown in Supplementary Figure S2. WHO-UNICEF estimates of national immunisation coverage show that only four EU/EEA countries, including Hungary, Portugal, Slovakia and Sweden, reported at least 95% coverage for both doses of MCV in 2017 [13]. Despite the estimated 99% measles vaccination coverage in Hungary [12, 14] (Supplementary Fig. S1), from February 2017 to July 2019, 70 measles cases were laboratory confirmed according to the European Centre for Disease Prevention and Control (ECDC) reports.

**Fig. 1.**
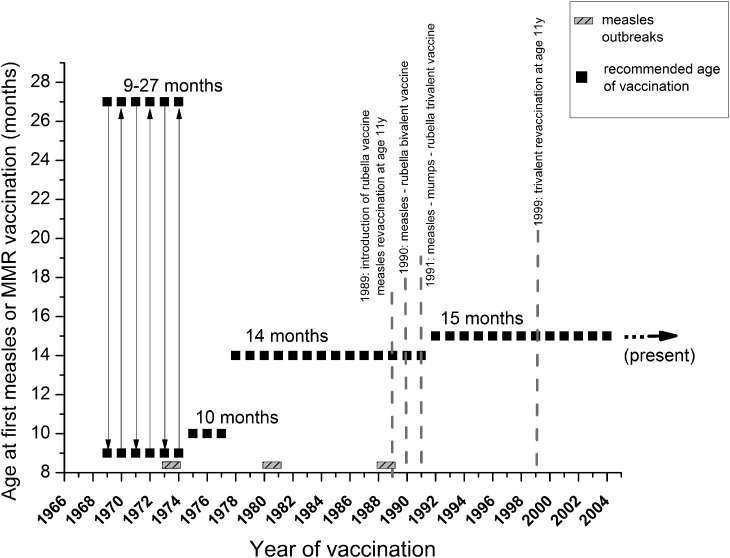
Measles and MMR vaccination schedules in Hungary. Serum samples were collected from all age groups (excluding neonates), and were categorised based on changes introduced in measles and MMR immunization schedules. Grey, shaded rectangles indicate measles outbreaks, black squares show the recommended age of the first dose of vaccine. Grey dotted lines mark the most important milestones of the vaccination schedule (introduction of reminder vaccines, changes between mono-, bi- and trivalent inoculum). Further details are described in Table 1.

These data raise the question concerning the reliability of the Hungarian population's herd immunity. Because of recent outbreaks worldwide, not only of measles, but also mumps and rubella (MMR) infections [15–19], and because of waning of immunity over time after vaccination [20–23], there is an urgent need for reliable and affordable laboratory tests for monitoring anti-MMR antibody (IgG) titres. For this purpose, we developed a new, ‘three-in-one’ immunoassay for quick measurement of all three anti-viral antibodies within a single run. To our knowledge, this triple format of MMR ELISA is currently not available on the market. The ELISA protocol described herein incorporates our previous method [24] that has been further improved to enable the use of the same assay conditions for all three anti-viral antibodies. We demonstrate the high-throughput applicability of this assay using 1736 serum samples from patients of diverse age groups, and provide an estimation of the population-level MMR seropositivity. We present and discuss our results in the context of both assay development and immunosurvey evaluation in relation to the history of M/MMR vaccination in Hungary from 1969 to present.

## Materials and methods

### Samples

A serum bank consisting of anonymous patient sera was established (*N*_total measles_ =3523 measles, *N*_mumps_ = 1736 mumps and *N*_rubella_ = 1736) from routine laboratory samples at the University of Pécs, Clinical Centre (Ethical License number 2015/5726). The samples are considered representative, as clinical residual samples were randomly selected (with the exclusion of seriously immunocompromised patients) from the Department of Laboratory Medicine, University of Pécs, Medical School, which serves three counties (Baranya, Somogy and Tolna, with a population of ~8 87 000), and receives laboratory examination requests from all over Hungary. In case of measles serosurvey, in order to give a more accurate estimate at population level, we combined our recently published data with the results of current measurements (previously we tested 1985 serum samples for measles [24], of which the data of 1787 samples have been pooled together with the current data; ‘cumulative’ data for measles, *N*_total measles_ = 3523). Serum samples were from all age groups (beginning from the era before the implementation of measles vaccine, through several different vaccine types, manufacturers and vaccination schedules, up to present), and were categorised based on past changes introduced in measles and MMR immunisation schedules (Table 1). The age group determination in our current study has been based on the landmarks in the history of measles and MMR vaccination schedules in Hungary, as detailed in Table 1.

**Table 1.** Age group categorization

Age groups	Explanation, rationale
	Vaccination groups were defined by adding the number of months indicated for the first childhood vaccine (e.g. 15 months of age) to the dates of birth. For example, a person born in February 1990 was assigned to age-group ‘*Patients vaccinated between 1991–1995*’, since this individual received the first measles (MMR) vaccine in May 1991
Patients born before 1969	Unvaccinated patients, wild-type infections. 1969: introduction of measles vaccine in Hungary (live, attenuated Leningrad-16 strain produced in the Soviet Union)
Patients vaccinated between 1969 and 1977	From 1969 to 1974, a single dose of measles vaccine was administered in mass campaigns to persons 9–27 months of age. The recommended age for vaccination was 10 months until 1978, when it was changed to 14 months. After the 1980–1981 epidemics, persons born between 1973 and 1977, who would have received vaccine when the recommended age was 10 months, were revaccinated. After 1989, children were re-vaccinated at the age of 11 years with monovalent measles vaccine in a scheduled manner. Consequently, the first individuals who received a reminder vaccine at the age of 11 were born in 1978. Thus, the cluster of 1969–1977 was the last that did not receive a reminder vaccine at the age of 11 as a part of the official vaccine schedule
Patients vaccinated between 1978 and 1987	These are the first individuals who benefited from the reminder monovalent measles vaccine at the age of 11. In 1999 the administration of trivalent vaccine was started in Hungary, consequently who received the first trivalent vaccine in 1999 were born in 1988
Patients vaccinated between 1988 and 1990	In 1989 the rubella vaccine was introduced, and the monovalent measles reminder vaccine at age 11 was started 1990: Introduction of measles–rubella bivalent vaccines
Patients vaccinated between 1991 and 1995	The administration of the first vaccine at age 14 months lasted from 1978 to 1991 1991: Measles–mumps–rubella trivalent vaccine 1992: MMR vaccine at age 15 months 1996: Introduction of MERCK MMR II – Enders’ Edmonston strain (live, attenuated)
Patients born between 1996 and 1998	1996: Introduction of MERCK MMR II – Enders’ Edmonston strain (live, attenuated) 1999: Measles–mumps–rubella re-vaccination (reminder shot) instead of monovalent measles vaccine 1999: Introduction of GSK PLUSERIX – Measles Schwarz Strain
Patients vaccinated between 1999 and 2002	1999: Introduction of GSK PLUSERIX – Measles Schwarz Strain 2003: Introduction of the GSK PRIORIX vaccine
Patients vaccinated in 2003	2003: Introduction of the GSK PRIORIX vaccine – attenuated Schwarz Measles
Patients vaccinated in 2004–2005	2004–2005: Administration of the MERCK MMR II
Patients vaccinated between 2006 and 2010	2006–2010 (5-year tender): GSK PRIORIX – attenuated Schwarz Measles
Patients vaccinated after 2011	Beginning from 2011 we use a Sanofi-MSD product; MMRvaxPro (Measles virus Enders’ Edmonston strain, live, attenuated) for vaccination and re-vaccination of children; GSK PRIORIX is still on the market, commonly used for vaccination in adulthood
Epidemics: 1973–74: Large epidemics, affecting primarily unvaccinated 6–9 years old children 1980–81: Another significant epidemic, affecting primarily 7–10 years old children 1988–89: Epidemic with high age-specific attack rates of 17–21 years old individuals, who had been vaccinated during the first years of the vaccination programme in Hungary 2017–18: Smaller epidemics with few connected and sporadic cases, derived mainly from virus importation	

Given the anonymous nature of samples, the only known data were the date of birth of the patients. Considering that we were interested in the differences between the various vaccination periods, dates of vaccination (instead of dates of birth) were chosen to define age group boundaries. By knowing the dates of birth and the important milestones of the Hungarian vaccination history (e.g. the first measles vaccine was introduced in Hungary in 1969; in 1990, the MR bivalent vaccines were introduced; and in 1991, the MMR trivalent vaccine was introduced; for further details, see Table 1), establishment of the vaccination-based age group matrix became feasible. Neonates and children under the age of vaccination were excluded from our study. As mentioned above, seriously immunocompromised patients were also excluded; however, patients with mild immunocompromised conditions may have been included.

### Antigen coating

**Fig. 2.**
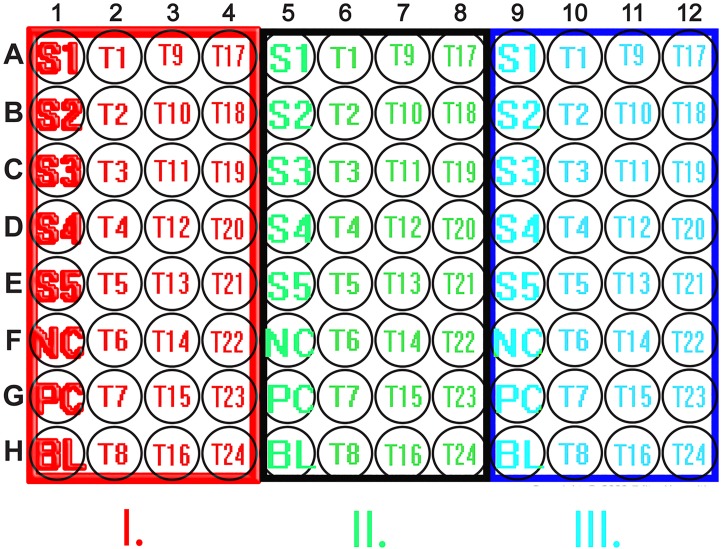
Schematic representation of the plate layout used for the ‘three-in-one’ ELISA. ELISA 96-well plates were divided lengthwise into three equal parts and each third was coated with the appropriate antigen. Assay parameters were optimised to enable equal conditions and common reagents for each antigen type. Abbreviations: (S1-S5) Standards; (PC) positive control;(NC) negative control; (BL) blank. (I) Measles antigen coating (measles virus, Edmonston strain); (II) Mumps antigen coating (mumps virus, Enders strain); (III) Rubella antigen coating (rubella virus, HPV-77 strain).

For our ‘target’ assay, we used purified, inactivated native virus preparations, derived from disrupted cells; measles Edmonston strain cultured in Vero cells (PIP013 Bio-Rad), mumps Enders strain cultured in BSC-1 cells (PIP014 Bio-Rad), rubella HPV-77 strain cultured in Vero cells (PIP044 Bio-Rad). Antigen preparations were sonicated before use, as per manufacturer's instruction. ELISA 96 well MicroWell™ MaxiSorp™flat-bottom 44-2404 plates (Nunc) were divided vertically into three equal parts and each third was incubated overnight at 4–6 °C with measles, mumps and rubella antigens (100 µl/well), respectively (Fig. 2, Table 2). Testing of blocking reagents was performed using bovine gelatine, milk powder, Block ACE (Bio-Rad) and our in-house polyvinyl alcohol (PVA)-based purely synthetic blocking buffer. Details of sample pre-treatment and assay preparation steps have been described earlier [24].

**Table 2.** Summary of major steps of the MMR indirect ELISA protocol

Coating antigen	Bio-Rad PIP013 Measles virus, Edmonston strain	Bio-Rad PIP014 Mumps virus, Enders strain	Bio-Rad PIP044 Rubella virus, HPV-77 strain
Concentration of the coating antigen used on microplates	2.8 µg/ml	3 µg/ml	0.4 µg/ml
Antigens are dissolved in ELISA Coating Buffer (Bio-Rad BUF030), overnight at 4–6 °C. Blocking ≥2 h, RT with our in-house purely synthetic blocking buffer			
Standard/quality control reagent (S1–S5)	3rd WHO International Standard for Anti-Measles (NIBSC code: 97/648)	Anti-Mumps Quality Control Reagent Sample 1 (NIBSC code: 15/B664)	Anti-Rubella Immunoglobulin 1st WHO International Standard Human (NIBSC code: RUBI-1-94)
Starting concentration of the standard/quality control reagent	~5000 mIU/ml	~1000 ‘Mumps Assay Unit’/ml, arbitrarily assigned	1600 International Units per ampoule
Negative control (NC)	A sample found to be negative in a previous run		
Positive control (PC)	A sample found to be positive in a previous run		
Incubation	3 × 15 min, 37 °C		
Colour detection	Polyclonal anti-human IgG HRP-conjugated (Dako polyclonal rabbit anti-human IgG or equivalent) + TMB		
Additional reagents	Washing Buffer (WB), used also for sample dilution in combination with the IgM Reducing Assay Diluent (Bio-Rad BUF038), as previously described (Böröcz *et al*. [24])		
Automation and reading	Siemens BEP 2000 Advance System, *λ* = 450/620 nm		

To demonstrate the lack of interference when using cell culture-derived antigen coatings, we compared our ‘target’ assay to purified recombinant viral capsid protein antigen-based assays. Purity of cell culture-derived, native virus-based coatings was verified by plate-to-plate comparisons to recombinant antigen-based coatings, as described below: ‘control’ microplates were coated with a series of doubling, four-point dilutions of recombinant antigens; measles virus Priorix, Schwarz strain nucleocapsid protein (Abcam ab74559, source: *Saccharomyces cerevisiae*) 1.66–0.207 µg/ml, mumps virus wild-type, Gloucester strain, nucleocapsid protein (Abcam ab74560, source: *S. cerevisiae*) 0.832–0.104 µg/ml, recombinant rubella virus capsid protein (Abcam ab43034, source: *Escherichia coli*) 2–0.25 µg/ml. To confirm the lack of interference by off-target molecules in whole virus-based assays, results of negative and low positive sample pools, international measles and rubella standards (3rd WHO International Standard for Anti-Measles, NIBSC code 97/648, Anti Rubella Immunoglobulin 1st WHO International Standard Human, NIBSC code RUBI-1-94), and the mumps quality control reagent (Anti-Mumps Quality Control Reagent Sample1) obtained for native virus-derived coatings were compared to the results obtained for different coating concentrations of recombinant antigens. Parallelism was tested to ascertain that the binding characteristic of the analyte (high and low antigen-titred sample pools) was the same, independent of the type of coating. For graphical representation, optical density (OD) values were linearised; dilution series of analytes were depicted as a function of common logarithm of both relative dilutions and OD values. Coating combinations with sufficiently high *R*^2^ values of the linear fittings (with the same slope) were selected for further analysis of correlation between ‘target’ and ‘control’ assays, using Bland–Altman plots.

### Cut-off

Determination of cut-off values was based on (a) Cohen's *κ* statistics, as an index of agreement between our assay and commercially available kits, (b) Area Under the Curve Receiver Operating Characteristics (AUROC) analysis (combined with Youden's *J* equation) – which in this case was used for comparing the performance of diagnostic tests [25] and (c) the ‘*experimental approach*’. The latter one was based on the mean OD and IU-transformed values yielded by our tests, belonging to selected serum samples that had been previously found negative by validated commercial kits. For assay testing, optimisation and comparisons, the following validated kits were used: measles IgG: Novalisa, Immunolab, Euroimmun, Sekisui-Virotech, Serion, Siemens Enzygnost; mumps IgG: Novalisa, Immunolab, Euroimmun, Sekisui-Virotech, Vircell; rubella IgG: Novatec, Immunolab, Euroimmun, DiaPro, Vircell.

Because our samples were anonymous (and consequently lacked clinical background), for the generation of AUROC curves, the establishment of the binary classifier system was based on averaged qualitative (positive, negative) results of commercial ELISAs.

In equivocal cases (and also to periodically check the assay performance), borderline and negative samples were measured using indirect immunofluorescence assays, using measles, mumps and rubella virus-infected cells, IIF (IgG) (Euroimmun). In case of commercial assays, calculation of qualitative results was performed according to default thresholds specified by the manufacturers. AUROC results were analysed using Youden's formula (*J* = sensitivity + specificity − 1), and the highest OD values were selected and transformed into units based on the standards (3rd WHO International Standard for Anti-Measles, Anti-Mumps Quality Control Reagent Sample 1, 1st WHO International Standard Human). For these transformations, sigmoid dose–response curves were fitted onto the dilution points of the standards.

### Analytical values, assay precision and specific assay characteristics

Analytical values such as lower limit of detection (LOD) and limit of quantification (LOQ) were determined by the mean and standard deviation of blank sample absorbance values; LOD was defined as mean + 3 s.d. and LOQ as mean + 10 s.d. (absorbance values), as suggested by the IUPAC Compendium of Chemical Terminology Gold Book. Sensitivity, specificity, positive and negative predictive values were also evaluated using validated commercial kits (Table 3).

**Table 3.** Assay precision and specific assay characteristics^a^

Specific assay characteristics (*N* = 474 from diverse age groups)	Measles	Mumps	Rubella
TPF = True Positive Fraction (sensitivity) = TP/(TP + FN)	0.99	0.99	0.99
FNF = False Negative Fraction (1–sensitivity) = FN/(TP + FN)	0.01	0.01	0.01
TNF = True Negative Fraction (specificity) = TN/(TN + FP)	0.93	0.94	0.88
FPF = False Positive Fraction (1–specificity) = FP/(TN + FP)	0.07	0.06	0.12
PPV = Positive Predicted Value = TP/(TP + FP)	0.99	0.99	0.99
NPV = Negative Predicted Value = TN/(TN + FN)	0.87	0.89	0.88
Intra-assay variability (CV%)^b^			
Positive sample 1	0.37	2.68	0.30
Positive sample 2	1.51	3.20	3.00
Positive sample 3	0.89	1.07	2.19
Negative sample 1	3.68	6.06	7.49
Negative sample 2	7.50	3.75	8.27
Negative sample 3	8.52	7.50	8.39
Inter-assay variability (CV%)^b^			
Positive sample 1	5.52	2.83	6.32
Positive sample 2	8.63	4.75	8.81
Positive sample 3	7.26	8.05	9.90
Negative sample 1	3.88	7.65	10.68
Negative sample 2	7.50	9.31	10.54
Negative sample 3	6.53	7.76	9.14

a Specific assay characteristics have been determined by comparing our assay to commercially available validated assays.

b Reproducibility, assay precision: intra-assay precision (coefficient of variation, CV%) was calculated for each of the three samples from the results of 12 determinations in a single run. Results for precision-within-assay are shown in the table above. Inter-assay precision (coefficient of variation, CV%) was calculated for each of the three samples from the results of three determinations in five different runs. Results for run-to-run precision are shown in the table above.

### Statistical analysis

AUROC analysis, Youden's *J* equation, confidence interval comparison at 95% confidence level (prop test) and Bland–Altman plot were used as statistical methods.

## Results

### Testing of antigen coating

**Fig. 3.**
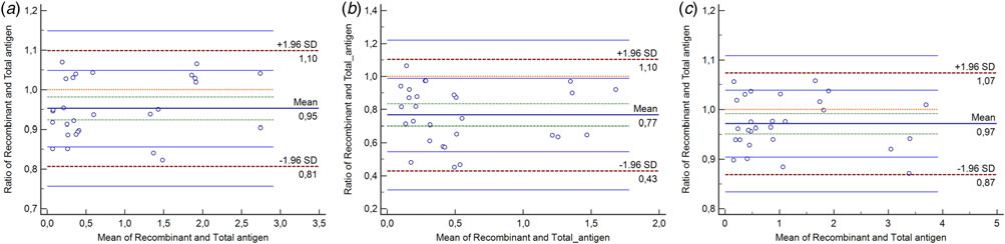
Comparison of whole virus *vs.* recombinant viral antigen-based ELISA coatings. Bland–Altman graphs display scatter diagrams of the ratios plotted against the averages of the two types of measurements. Sample number = 28 (duplicates of the dilution series of positive and negative sample pools and quadruplicates of the dilution series of standards). Limits of agreement (LoA) are defined as the mean difference ± 1.96 s.d. (95% confidence interval). Since data points do not exceed the maximum allowed difference between methods (dotted brown lines), and no pronounced trend is observable, the two methods (target: total antigen repertoire-based coating *vs.* control: recombinant antigen-based coating) are in agreement and can be used interchangeably. Recombinant antigen coatings: Measles virus Priorix, Schwarz strain nucleocapsid protein, Mumps virus wild-type, Gloucester strain nucleocapsid protein, Recombinant Rubella virus nucleocapsid protein. Optimal recombinant antigen- based concentrations: 0.83 µg/mL, 0.416 µg/mL, 1.0 µg/mL for measles, mumps and rubella, respectively. Optimal inactivated pathogen-based coating concentrations: 2.8 µg/mL, 3.0 µg/mL, 0.4 µg/mL for measles, mumps and rubella, respectively. Sample number (n): N=28 (Samples were used in duplicates, standards were used in quadruplicates).

To check whether the entire virus-based coatings (derived from cell cultures) contain off-target molecules, we compared our assays to purified recombinant viral capsid protein antigen-based (in-house) assays. Based on the linearity tests, the following recombinant viral nucleocapsid antigen coatings were selected: measles 0.83 µg/ml, mumps 0.416 µg/ml and rubella 1.0 µg/ml (*R*^2^ standards ≥0.97, *R*^2^ samples ≥0.93) (Supplementary Fig. S3). Bland–Altman plots were then generated; ratios of the results from the two techniques (‘target’ *vs.* ‘control’ assay) were plotted against the averages. As shown in Figure 3, we obtained data points that fell within the range ±1.96 s.d. (confidence interval 95%), with no observable trends, suggesting that the two methods are in agreement, thus demonstrating the adequate purity of the entire virus-based coating system used in the ‘target’ assay.

### Cut-off determination and assay precision

**Fig. 4.**
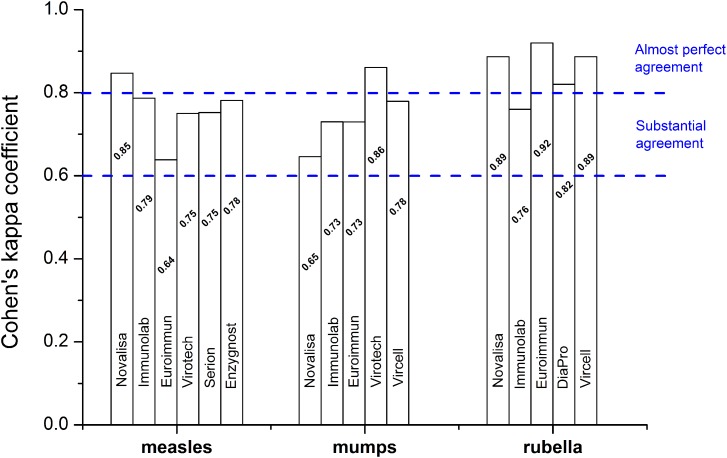
Cohen's *κ* analysis of plate-to-plate measurements (*N*_Novalisa, Immunolab, Vircell_ = 84, *N*_Virotech, DiaPro_ = 80, *N*_Euroimmun_ = 88, *N*_Serion, Enzygnost_ = 90 samples). The measures of agreement describing the inter-rater reliability varied between ‘substantial’ and ‘almost perfect’.

**Fig. 5.**
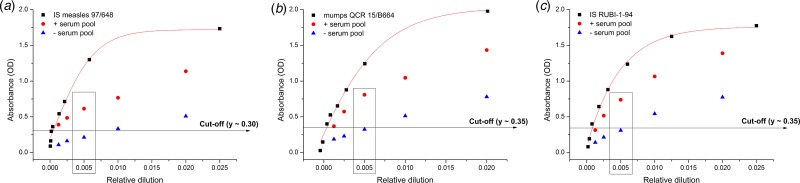
Typical standard curves of MMR assay. Sigmoid dose–response curves of the dilution series of the standards were generated with optimal data fitting (*R*^2^ ≥ 0.97). Absorbance values are plotted in function of relative dilution (1/dilution). These curves serve as the base for the conversion of OD values to units/ml. Rectangles show the optimal serum dilutions (200-fold) used in the final assay format. Model and equation used for calibration curve: sigmoid dose-response curve; y = A1 + (A2-A1)/ (1 + 10^ ((LOGx0-x)*p)). Adjusted R^2^ values: 0.97, 0.97, 0.99 for measles, mumps and rubella, respectively. Measurement ranges: 0.025–12.5 mIU/mL, 0.02–10.0 arbitrary U/mL, 2.0 – 265 mU/mL for measles, mumps and rubella, respectively. Cut-off values: 0.15 mIU/mL, 0.15 arbitrary U/mL, 9.5 mIU/mL. LOD (mean + 3SD) extinction (OD) values: 0.08, 0.10, 0.08 for measles, mumps and rubella, respectively. LOQ (mean + 10SD) extinction (OD) values: 0.20, 0.23, 0.20 for measles, mumps and rubella, respectively.

Cohen's *κ* analysis was performed; plate-to-plate *κ* statistics (using tests described in the Materials and methods section) gave ‘substantial’ to ‘near-perfect’ agreement; 0.64 ⩽ *κ* ⩽ 0.92 (Fig. 4). AUROC areas were ≥0.92, for all three antigens (Supplementary Fig. S4). Based on the AUROC analysis, with the help of Youden's equation, the following sensitivity–specificity pairs were selected 0.985–0.975, 0.935–0.911, 0.989–0.946 for measles, mumps and rubella, respectively. According to the ‘experimental approach’, cut-off values were set for all antigen types (measles, mumps, rubella) based on mean observed OD values belonging to diagnostically seronegative samples (3 × 15 samples, OD_negative sample_ ⩽ 0.28, 0.37, 0.34 for measles, mumps and rubella, respectively; data not shown). Cut-off values calculated based on empirical results were concordant with the statistically computed values. Typical dose–response curves obtained for measles, mumps and rubella standards are shown in Figure 5. Analytical values, such as lower LOD and LOQ are also represented in Figure 5. Sensitivity, specificity, positive and negative predictive values are shown in Table 3. We selected randomly chosen negative samples from the measles, mumps and rubella groups (30 each) that were verified using indirect immunofluorescent microscopy. We found 93%, 90% and 96% correspondence for measles, mumps and rubella, respectively (data not shown).

### Assay characteristics: cost, ease and time requirement

**Fig. 6.**
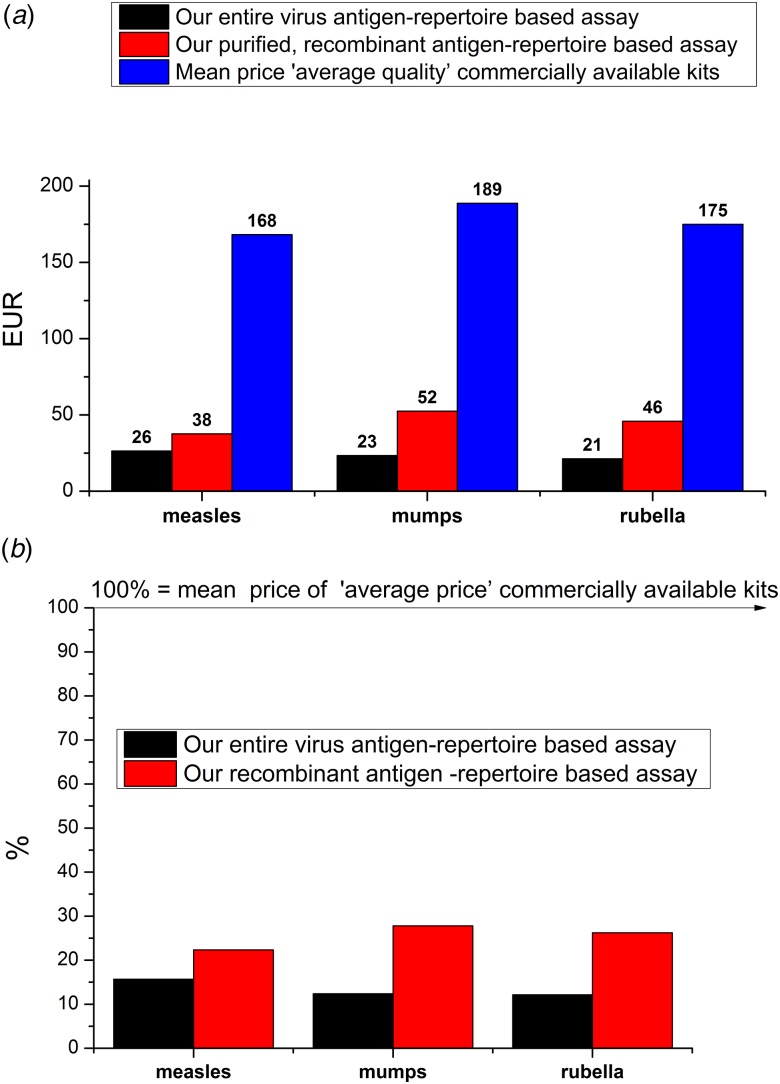
(a) Comparison of assay prices (commercial kits) and costs (our test) expressed in Euros. (b) Ratios of assay prices: ‘average price’ commercial kits *vs.* our test expressed in percentages. The average price of commercial kits was calculated based on the Hungarian distributor prices (VAT included), and included only those assays that we applied during the optimization and the test-to-test comparisons (Materials and methods section). Siemens Enzygnost assays – belonging to a higher price-range – were excluded from the calculation.

**Fig. 7.**
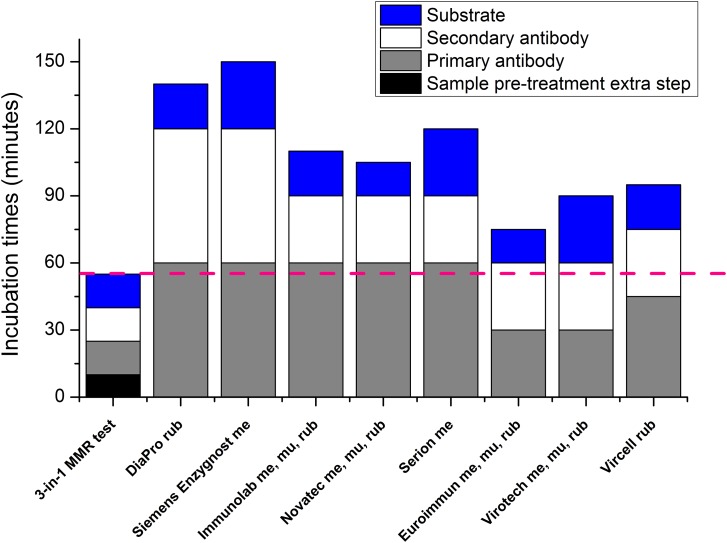
Comparison of incubation times of our test (three-in-one MMR) to different commercial kits (me = measles, mu = mumps, rub = rubella).

An important feature of our three-in-one MMR ELISA assay is affordability; it costs only a fraction of the commercially available assays (Fig. 6a, b). An important component for improving the signal-to-noise ratio (background reduction) is a self-developed, low-cost reagent, a protein-free PVA-based blocking buffer (Supplementary Fig. S5). Another important feature is the reduced assay duration time; compared to the ~1.5/2.5 h of timeframe of commercially available tests (used for parallel and justificatory measurements), our test can be performed within 1 h (Fig. 7).

### Determination of age groups with highest frequencies of seronegativity

**Fig. 8.**
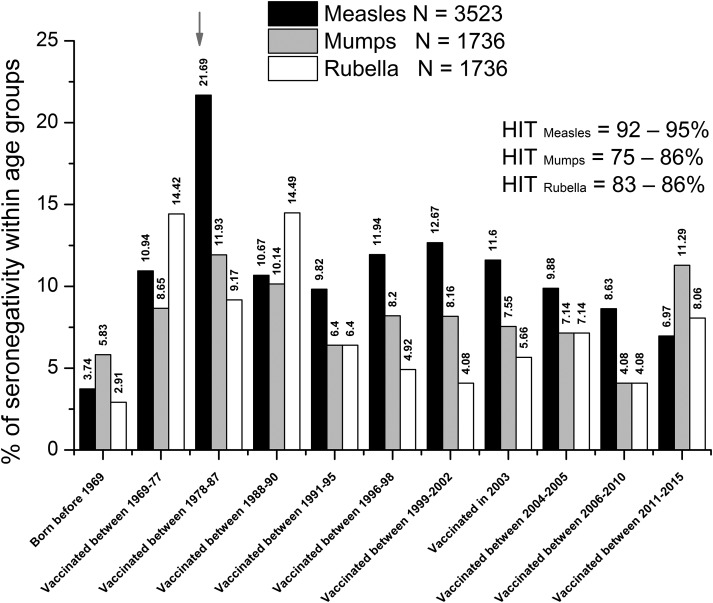
Vaccination period-independent summary of results. Considering the age-independent totality of samples, the anti-measles, mumps and rubella IgG antibody titres were inadequate in 10.16%, 8.18% and 7.72%, respectively. Considering HIT values, population-level seropositivity ratio of anti-measles antibodies failed to reach the criteria for herd immunity (seropositivity ≥95%). The red arrow shows the vaccination group with highest seronegativity in terms of anti-measles antibody titers.

**Fig. 9.**
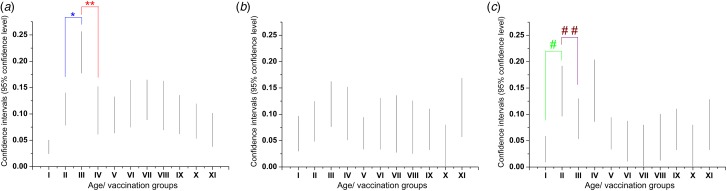
Summary of seronegativity ratios within different age groups. Age or vaccination groups (X-axis): (I) Born before 1969 (not vaccinated; high probability of wild-type infection), (II) vaccinated between 1969-77, (III) vaccinated between 1978-87, (IV) vaccinated between 1988-90, (V) vaccinated between 1991-95, (VI) vaccinated between 1996-98, (VII) vaccinated between 1999-2002, (VIII) vaccinated in 2003, (IX) vaccinated between 2004-2005, (X) vaccinated between 2006-2010, (XI) Vaccinated between 2011-2015. P-values indicating statistically significant differences between adjacent age groups: (*) vaccinated between 1969-77 and 1978-87 p=0.00003841; (* *) vaccinated between 1978-1987 and 1988-90 p=0.0015; (#) vaccinated between 1969-77 and 1978-87 p=0.00008437; (##) vaccinated between 1988-90 and 1991-95 p=0.008532. We identified samples in the cluster ‘*Vaccinated between 1978–1987*’ as the lowest seropositivity group for measles.

**Fig. 10.**
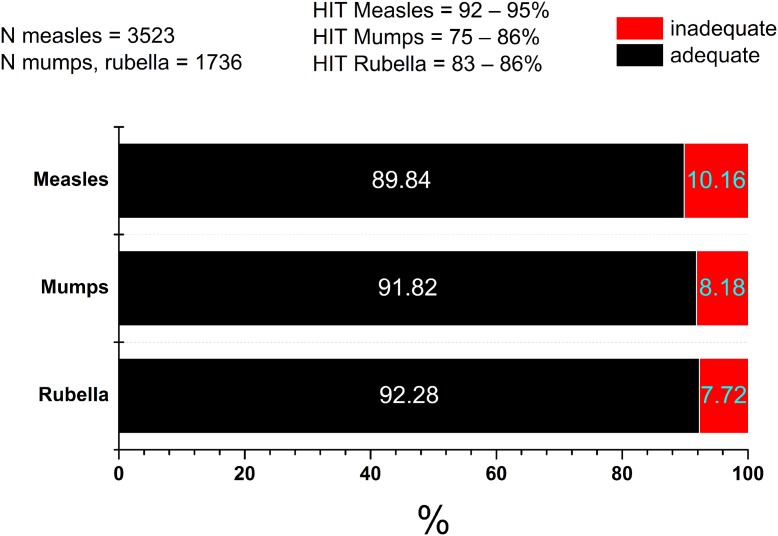
Vaccination period-dependent confidence intervals of seronegativity. Relative frequencies of measles-, mumps- and rubella-specific seronegativity dependent on the period of vaccination. Vertical lines indicate 95% confidence intervals. Significant differences between the antibody levels of the critical age groups and their flanking age groups are marked with asterisks.

Considering the antigen-specific seropositivity ratios of all samples measured, anti-measles, -mumps and -rubella IgG antibody titres were adequate in 89.84%, 91.82% and 92.28%, respectively (Fig. 8). Taking the following herd immunity threshold (HIT) values as a base; HIT_Measles_ = 92–95%, HIT_Mumps_ = 75–86%, HIT_Rubella_ = 83–86, it can be stated that regarding measles, levels of humoral immunity may be inadequate in certain age clusters of the population. Regarding anti-measles antibodies, cumulative data (*N*_total measles_ = 3523 serum samples) show that the lowest seropositivity (79.3%) was detected in individuals vaccinated between 1978 and 1987 (Figs 9 and 10), with significant differences from the flanking age groups vaccinated between 1969–1977 and 1988–1990 (*P* = 0.00004 and *P* = 0.0015, respectively) (Fig. 10). For mumps (*N* = 1736 serum samples), the least protected groups were vaccinated during 1978–1987 (11.9%) and 1988–1990 (10.1%); however, these were not statistically different from the adjacent age groups. In the case of rubella (*N* = 1736 serum samples), the least protected groups were vaccinated during 1969–1977 (14.4%) and 1978–1987 (14.5%). Significant differences were observed between the group born before 1969 (not vaccinated) and vaccinated during 1969–1977 (*P* = 0.00008), and between groups 1988–1990 and 1991–1995 (*P* = 0.009).

## Discussion

Regarding assay optimisation, an important requirement was the equalisation of incubation times used in the three-in-one MMR ELISA. The establishment of a combined test system using identical serum dilutions, reagent volumes and incubation times that enable the measurement of 24 samples for all three antigens within a single run was only feasible with the maximal reduction of potentially interfering factors. An important step was the reduction of non-specific signal by using an IgM reducing assay diluent (Bio-Rad). The second important step was the use of our in-house PVA-based synthetic blocking buffer that enabled an optimal signal-to-noise ratio at a minimal cost. These steps made it possible to use a high concentration of antigen coatings, which in turn allowed relatively short incubation times and high performance of our assay.

As mentioned in the Introduction section, in Hungary, between February 2017 and July 2019, 70 measles cases were laboratory confirmed according to ECDC reports [5]. During the epidemics of 2017, there have been 36 measles cases in Hungary (five imported, 26 import-related, four unknown/not reported and one endemic). Regarding the infections by age group and vaccination status for 2017, according to WHO data, among the individuals 20–29 years of age, ≈35% had not been vaccinated, and ≈65% received two or more doses of vaccine. Of individuals older than 30 years, ≈18% had not been vaccinated, ≈24% received one dose, ≈26% received two or more doses of vaccine and ≈32% were of unknown vaccination status. Based on these data, it can be hypothesised that in the case of vaccinated adults (≥20 years of age), who had received two or more doses of vaccine, vaccine insufficiency may have underlaid the infections [12]. The last case of parotitis epidemica reported in Hungary was a non-vaccinated 35-year-old man, who became infected during the summer of 2018. Between 2012 and 2016, Hungary reported 21 mumps cases [12, 26]. In the 2007 local mumps outbreak, the epidemic started from an individual who returned home from Ukraine in December 2007. Soon after his case, individuals from his personal connections became affected (later all nine cases were laboratory confirmed). Previously, in 2003, comparably small outbreaks were reported in close communities of unvaccinated people (student houses, schools) [11]. Considering rubella in Hungary, between 2007 and 2018, WHO reported only 10 known cases [12]. This is a significant development compared to the end of 1990s and beginning of 2000s, when ~100 rubella cases per year were reported (WHO statistics). By 2006, this number decreased by 80% (22 reported cases in year 2006). Measles, mumps and rubella statistics (cases per year) are shown in Supplementary Figure S2.

In 1969, the measles vaccine was introduced in Hungary in the form of live, attenuated Leningrad-16 strain vaccine, produced in the former Soviet Union. Between 1969 and 1974, a single dose of vaccine was administered in campaigns to individuals of 9–27 months [5]. After vaccination was implemented, the incidence rate decreased until 1973–1974, when large epidemics occurred primarily in unvaccinated 6–9 years old [5], questioning the effectiveness of the early vaccination programme. Regarding post-vaccination humoral immune response, heterogeneous data are available in the literature. It is generally accepted that the success of vaccination in children is dependent on the presence (or absence) of inhibitory maternal antibodies and the immunologic maturity of the recipient, as well as on the dose and vaccine strain. It is also recognised that the age of ≥12 months is a milestone in the development of an efficient immune response. A 2015 meta-analysis based on WHO study published the following seroconversion rates: 50% (95% CI 29–71%) at age 4 months, 67% (95% CI 51–81%) at 5 months, 76% (95% CI 71–82%) at 6 months, 72% (95% CI 56–87%) at 7 months and 85% (69–97%) at 8 months. Interestingly, the likelihood of seroconversion in children depends not only on the child's age, but also on the age of the mother; older children generally respond better than younger children, and children of younger mothers have the tendency to respond better than children of older mothers. Moreover, the ‘source’ of the mother's immunity (disease- or vaccination-induced) also plays a role as a surrogate factor [27]. The current Advisory Committee on Immunization Practices (ACIP 2012) also recommends age ≥12 months for the first MMR vaccination. As a general rule, the optimal vaccination age should be defined by the dynamics of the age-dependent progress in seroconversion, balanced by the level of the epidemiological risk [28, 29]. According to the Hungarian vaccination practice, the MMR vaccine is given twice; at 15 months and 11 years of age.

Regarding immunocompromised individuals and children with contraindications, we would like to note that in Hungary, immunocompromised persons also complete the recommended immunisation series against vaccine preventable diseases, whenever possible. The vaccination practice follows international guidelines (2013 IDSA), and an individualised patient approach is applied. This implies the involvement of a vaccination expert who performs case-to-case risk evaluation. As a general rule, live viral vaccines (e.g. polio, MMR, varicella) that may induce severe systemic reactions in immunocompromised individuals should not be administered to patients with severe immunosuppression and/or immune deficiency. Nevertheless, important exceptions exist: certain live vaccines can be administered in some immune system disorders or when the benefit of the vaccine outweighs the side effects, or major risk arising from the epidemiological environment [30, 31].

Our current serological data are in agreement with our previous report [24] where the estimated seropositivity for cluster ‘1978–1987’ was ~74.6%, followed by cluster ‘1969–1977’ with ~84.6%. A recent publication by Hungarian colleagues has reported 86.2% seropositivity for the 41–45 years old individuals [32], a cluster partially overlapping with the two abovementioned age groups of our classification. The potential gap detected in herd immunity is also supported by the already known insufficiencies during the initial vaccination era [33]. These individuals were vaccinated during the early 1970s, when the optimal age of vaccination was not well defined, and the thermolability of the reconstituted vaccine was not fully characterised [5]. These relatively high measles seronegativity ratios may have been a consequence of vaccine inefficiency, which seems to be supported by historical data: after the starting of the immunisation campaign in 1969, a decade later, in 1978, the recommended age for vaccination was changed from 10 to 15 months. The 1988–1989 epidemics affected individuals (16–22 years old) who were vaccinated in the early era with a singular measles vaccine. Following the 1988–1989 epidemics, persons born between 1973 and 1977 were revaccinated [33]. Even though a significant portion of the age groups indicated with ** in Figure 9 later were re-vaccinated or contracted wild-type measles infection (and thus mounted high IgG antibody response), in this cluster, we found the lowest cumulative anti-measles antibody titres (i.e. high ratio of seronegativity), which suggests the ineffectiveness of the early vaccination system. Additional support for this hypothesis is the high age-specific attack rates during the 1988–1989 outbreak that affected the population with ≥93% vaccine coverage. After the introduction of the trivalent MMR vaccine (1991), we detected a statistically significant improvement in the anti-measles antibody titres (Fig. 10). The group ‘*Vaccinated between 1988–1990*’ has significantly better humoral response compared to the group ‘*Vaccinated between 1978–1987*’, reflecting the effectiveness of the trivalent reminder vaccine at age 11.

Population-level result evaluation was performed in relation to the concept of herd immunity. The term ‘herd immunity’ is widely used, but diversely interpreted. We used it in the sense of ‘a threshold proportion of immune individuals’ [34], strictly limited to humoral antibody titres. This threshold denoting the arrest of disease spread is different for every disease and is affected by many factors; key epidemiological parameters, such as the age-specific force of infection and the basic reproduction number (*R*_0_) are estimated from case notification or serological data [35]. Imperfect immunity (due to individual differences of responders), heterogeneous populations with potential non-random mixing and non-random vaccination schedules may also need to be considered [34]. *R*_0_ is defined as the average number of secondary cases that result from an individual infection in a susceptible population [36]. Estimates of *R*_0_ depend on underlying mixing assumptions. For the virus-specific *R*_0_ values shown below, the model of ‘likely mixing patterns’ was used [35]. The *R*_0_ estimates are highest for measles, intermediate for mumps and generally lowest for rubella [35]. For measles, *R*_0_ is often cited as 12–18, which implies the need for ~95% herd immunity. This means that each person with measles can infect 12–18 other individuals in a completely susceptible population. For this reason, the achievement of ≥95% of immunity across all age groups (optimal immune response followed by efficient seroconversion on population level) is the official target for measles elimination. In the literature, *R*_0_ and HIT values are generally estimated as follows: *R*_0 Measles_ = 12–18, HIT_Measles_ = 92–95%, *R*_0Mumps_ = 4–12, HIT_Mumps_ = 75–86%, *R*_0Rubella_ = 5–7, HIT_Rubella_ = 83–86% [35]. Often used models for population-level estimation are the HIT (*I*_c_); *I*_c_  =  1 − (1/*R*_0_), and the critical vaccination coverage (*V*_c_); *V*_c_  =  *I*_c_/*E*, where *E* is vaccine effectiveness [34–37]. Despite the remarkable theoretical knowledge, public health practice aims at 100% coverage, with all the doses recommended, bearing in mind that – because of the diversity of individual immune responses – 100% is never achievable.

## Limitations

We would like to note that our ‘*three-in-one*’ assay and the results described in our paper may have certain limitations. As specified in the WHO *Manual for the Laboratory-based Surveillance of Measles, Rubella, and Congenital Rubella Syndrome*, EIA/ELISA testing may be used for the detection of the presence (or absence) of anti-viral IgG antibodies of individuals, as well as to perform population-level immunity estimations. In case of population-based seroprevalence studies, ELISA/EIA results can help characterise the immune profile of target populations; however, there are important limitations. When applying commercial assays, we used cut-offs and calculation methods as per kit manual, without changing or reinterpreting default thresholds. Each commercially available kit (listed in Materials and methods section) specified one particular method for quantitative (and qualitative) result calculation, with no distinction between periods with or without epidemics, or samples collected with the purpose of clinical diagnosis or population-level survey. However, according to the literature, thresholds for commercial IgG ELISAs/EIAs were determined with the purpose of individual patient management, rather than with population-level antibody prevalence calculations [38, 39]. A positive result of virus-specific IgG clearly demonstrates an immune response, in contrast, given that commercial immunoassays are capable only of humoral antibody detection, a negative or equivocal result cannot be considered as an absolute proof for lack of immunity [40, 41]. The functional characteristics and the ‘quantity’ of antibodies may be highly correlated with protection or synergistic with other functions (e.g. with cellular immunity). The correlates of vaccine-induced immunity are often a matter of debates; for some vaccines, we have no true correlates, but only useful surrogates [42, 43]. As far as Plotkin's definitions are considered normative [44], entire antigen repertoire-based ELISA/EIA methods of measles, mumps and rubella IgG antibody detection are considered rather than a good surrogate marker for immunity. This is especially true for our test, since our cut-off calibration was based on multiple measurements with independent, commercially available assays, and with indirect immunofluorescent microscopy. The diagnostic ability of our test is calculated based on the results obtained by kits capable of humoral antibody detection, and not on neutralising antibody titres that could serve as an absolute correlate of protection.

Additionally, considering age-specific susceptibility estimates at population level, the phenomenon of vaccination-induced lower antibody levels, compared to those elicited by natural infection, is also to be taken into account [45, 46]. Consequently, low (negative or equivocal) IgG results are to be interpreted with caution, when assessing immunity in populations with effective immunisation programmes [38]. The evaluation of immune status may require additional testing of specimens with results in the equivocal range (we used IIF for this purpose). We also would like to note that the actual level of any immunological marker is a snapshot in time, which needs to be interpreted in the light of the kinetics of the marker. Although the half-lives of antibodies against measles, mumps and rubella are relatively long, unexpected responses cannot be excluded, whereby vaccinees can mount sufficient responses rapidly from a low (even close to zero) background of humoral antibody level [44].

## Conclusions

Here we describe the development of a time-saving, cost-effective and standardised immunoserological assay for simultaneous detection of anti-measles, -mumps and -rubella IgG antibodies in human sera. The importance of the ‘three-in-one’ assay is highlighted by recent outbreaks of measles, mumps and rubella infections in several countries. This triple assay is based on an operation protocol that uses the same reagent load with uniform, short incubation times and equally pre-treated samples, enabling the three-parametric screening of 24 samples per plate within 1 h. In high-throughput automated settings, separate testing of the three antigen types is also feasible, thus allowing the measurement of 80 samples per run. Considering the HIT values, anti-measles seropositivity (79.3%) of the ‘1978–1987’ vaccination group suggests the existence of potential gaps in anti-measles immunity of the population. For mumps and rubella, our preliminary data suggest satisfactory immunity levels. The potential gaps in anti-measles immunity warrant further large-scale serological screening.

## Supplementary material

For supplementary material accompanying this paper visit https://doi.org/10.1017/S0950268819002280. click here to view supplementary material
